# The initial CT blend sign is not associated with poor patient outcomes after stereotactic minimally invasive surgery

**DOI:** 10.1186/s12883-021-02181-0

**Published:** 2021-04-15

**Authors:** Xu Yang, Yan Zhu, Linshan Zhang, Likun Wang, Yuanhong Mao, Yinghui Li, Jinbiao Luo, Guofeng Wu

**Affiliations:** 1 Emergency Department of Affiliated Hospital, Guizhou Medical University, No. 28, Guiyijie Road, Guiyang City, 550004 Guizhou Province People’s Republic of China; 2 Department of Neurological Rehabilitation, Shanghai Second Rehabilitation Hospital, No. 25, Lane 860, Changjiang Road, Songnan Town, Baoshan District Shanghai, 200441 People’s Republic of China; 3 Department of Neurosurgery, Guangzhou First Peoples’ Hospital, No. 1, Panfu Road, Guangzhou City, 510000 Guangdong Province People’s Republic of China

**Keywords:** Intracerebral haemorrhage, Stereotactic minimally invasive surgery, Glasgow coma scale, National Institute of health stroke scale. Blend signs

## Abstract

**Background:**

The initial CT blend sign is an imaging marker that has been used to predict haematoma expansion and poor outcomes in patients with small-volume intracerebral haemorrhage (ICH). However, the association of the blend sign with the outcomes of patients undergoing surgery remains unclear. The present study aimed to retrospectively evaluate the influence of the initial CT blend sign on short-term outcomes in patients with hypertensive ICH after stereotactic minimally invasive surgery (sMIS).

**Methods:**

We enrolled 242 patients with spontaneous ICH. The patients were assigned to the blend sign group (91 patients) or non-blend sign (control) group (151 patients) based on the initial CT features. The NIHSS, GCS and mRS were used to assess the effects of sMIS. The rates of severe pulmonary infection and cardiac complications were also compared between the two groups.

**Results:**

Statistically significant differences in the NIHSS and GCS scores were not observed between the blend sign group and the control group. No significant differences in the proportion of patients with good outcomes during the follow-up period were observed between the two groups. A higher rate of re-haemorrhage was noted in the blend sign group. Significant differences in the rates of severe pulmonary infection and cardiac complications were not observed between the two groups.

**Conclusions:**

The initial CT blend sign is not associated with poor outcomes in patients with hypertensive ICH after sMIS. ICH patients with the CT blend sign should undergo sMIS if they are suitable candidates for surgery.

## Background

Spontaneous ICH is a life-threatening disease associated with high global mortality and morbidity rates worldwide. Various clinical trials on ICH treatment have been performed within the past 10 years to improve patient outcomes [1]. However, although many clinical trials on ICH therapy have been performed, the death rate at 30 days remains as high as 40% worldwide [2]. No therapeutic strategies have been proven to be effective in improving functional outcomes [3]. Open surgery for removing ICH was not more advantageous than medications for patients with ICH in several trials [4]. Although craniotomy can remove ICH effectively, it can result in severe injuries to the brain and severe pulmonary infection [4]. The benefits of open surgical procedures over conservative medications for hypertensive ICH remain disputed [5]. Early neurosurgical procedures for ICH treatment have not been shown to be more beneficial than initial conservative treatment [6]. Brain injuries caused by conventional surgical procedures might counteract the potential advantages of haematoma removal during open surgery [7]. In recent years, sMIS for ICH treatment has been assessed in several clinical trials and has demonstrated promising results [8–10]. Minimally invasive punctures and drainage have been shown to cause the least damage to the brain and lead to the shortest operative time [4]. In a recent study, although MIS followed by thrombolysis for ICH treatment did not lead to a higher proportion of patients with good functional outcomes, haematomas that decreased to 15 ml or less led to improved mRS scores at the end of the follow-up period in stabilized patients [4].

Haematoma growth (HE) indicates a very poor outcome. HE may be prevented if physicians can identify patients with high-risk factors in the early stage of ICH [2]. Some imaging markers, such as the blend sign, black hole sign and spot sign on brain CT, have been recognized as predictive factors for HE [2, 8–10]. The initial CT blend sign has been reported to be associated with poor outcomes in patients with ICH following the administration of medications [2]. The CT black hole sign and the blend sign can predict postoperative re-haemorrhage after sMIS [11, 12]. However, whether the initial CT blend sign is associated with poor outcomes in patients following sMIS remains unknown. We speculated that the initial CT blend sign is associated with poor outcomes in patients with ICH after sMIS. The present study aimed to retrospectively determine the influence of the initial CT blend sign on outcomes in patients with spontaneous ICH following sMIS.

## Methods

The clinical data of patients with ICH who underwent sMIS in the Affiliated Hospital of Guizhou Medical University were collected and analysed. The recruitment period was from January 1, 2018 to June 30, 2019.

### Study design and participants

#### Study design

The study was performed retrospectively to determine the relationship between the initial CT blend sign and poor outcomes in patients with ICH after sMIS. The clinical data from patients with ICH were retrieved from medical records. The diagnosis of ICH was confirmed by using a baseline CT scan taken within 1 h of admission. The patients with ICH were assigned to the blend sign group or non-blend sign group (control group) based on the initial CT scan. sMIS was performed within 27 h of admission.

The inclusion criteria were the same as those in our previously published clinical studies [12]. In brief, patients who met the diagnostic criteria for ICH based on non-enhanced CT scans were included. ICH was located in the supratentorial area. The ICH volume was 30ml-50ml. Consent for surgery was obtained from the patients’ authorized representatives.

The exclusion criteria were the same as those in previously published studies [12]. Patients with ICH located in the brainstem and cerebellum were excluded from this study. Patients with secondary ICH due to haemorrhagic transformation from brain infarction were also excluded.

#### Participants

**Fig. 1:**
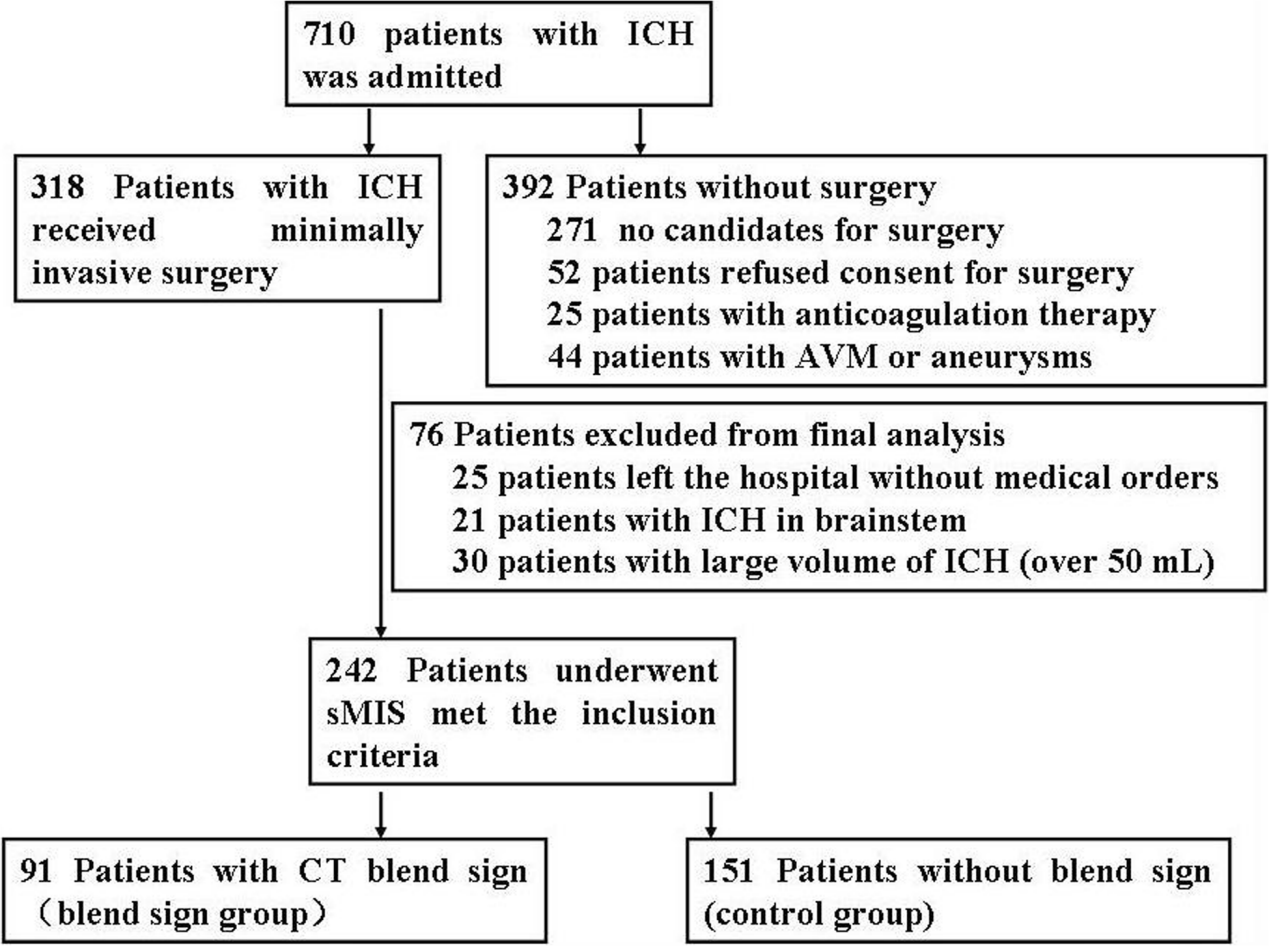
Flowchart of patients for selecting candidates for minimally invasive surger. A total of 710 patients with ICH were admitted. Three hundreds and eighteen patients received stereotactic minimally invasive surgery. Finally, only 242 patients with ICH met the inclusion criteria

A total of 710 patients with spontaneous ICH were admitted to our hospital during the study period. Among them, 318 patients with ICH were surgical candidates and underwent sMIS. However, 25 patients with ICH left the hospital within 1 week without medical orders, 21 patients developed ICH in the brainstem, and another 30 patients had ICH with a volume over 50 mL. These 76 patients were excluded from the final analysis (Fig. 1).

Based on the inclusion criteria, 242 consecutive patients who underwent sMIS were included in the present study. The blend sign group included 91 patients, and the non-blend sign group (control group) included 151 patients with spontaneous ICH. The baseline clinical characteristics of the patients are listed in Table 1.

**Table 1:** Baseline data between blend sign group and control group

Factors	Blend sign group(91)	Control group(151)	*P*-value
Ages (years, ^−^x ± SD)	56.18 ± 12.61	57.58 ± 12.77	0.416
Sex (male, %)	73 (80.2%)	107 (70.9)	0.106
History of smoking(n, %)	46 (50.5%)	75 (49.7%)	0.500
History of drinking(n, %)	41 (45.1%)	67 (46.9%)	0.447
History of hypertension(n, %)	68 (74.7%)	110 (56.7%)	0.004
Anticoagulants(n, %)	2 (2,2%)	4 (2.6%)	0.594
History of diabetes(n, %)	2 (2.2%)	10 (6.4%)	0.119
Haematoma volume (ml, IQR)	37.8 (33–52.5)	38 (31–50)	0.379
Systolic pressure (mmHg, ^−^x ± SD)	174.03 ± 24.96	173.33 ± 29.53	0.844
Diastolic pressure (mmHg, ^−^x ± SD)	103.75 ± 15.67	100.63 ± 21.70	0.198
GCS on admission (points, IQR)	11 (8–13)	11 (7–13)	0.583
NIHSS on admission (points, IQR)	16 (14–19)	14 (16–21)	0.304
Time from onset to admission (hour, IQR)	5 (3–10)	6 (2–9)	0.802
Time from onset to baseline CT(h, IQR)	4 (2–8)	5 (2.5–10)	0.163
Time from admission to surgery(h, IQR)	15 (9–27)	15 (9.8–27)	0.466
Duration of surgery(h, IQR)	1.4 (1.0–1.9)	1.5 (1.0–2.0)	0.130
Time for removing the tube (days, IQR)	4 (2–6)	4 (3–6)	0.904
Good outcome (n, %)	50 (54.9)	78 (51.7)	0.619

*GCS* Glasgow Coma Scale, *NIHSS* National Institute of Health Stroke Scale

### Imaging analysis

**Fig. 2:**
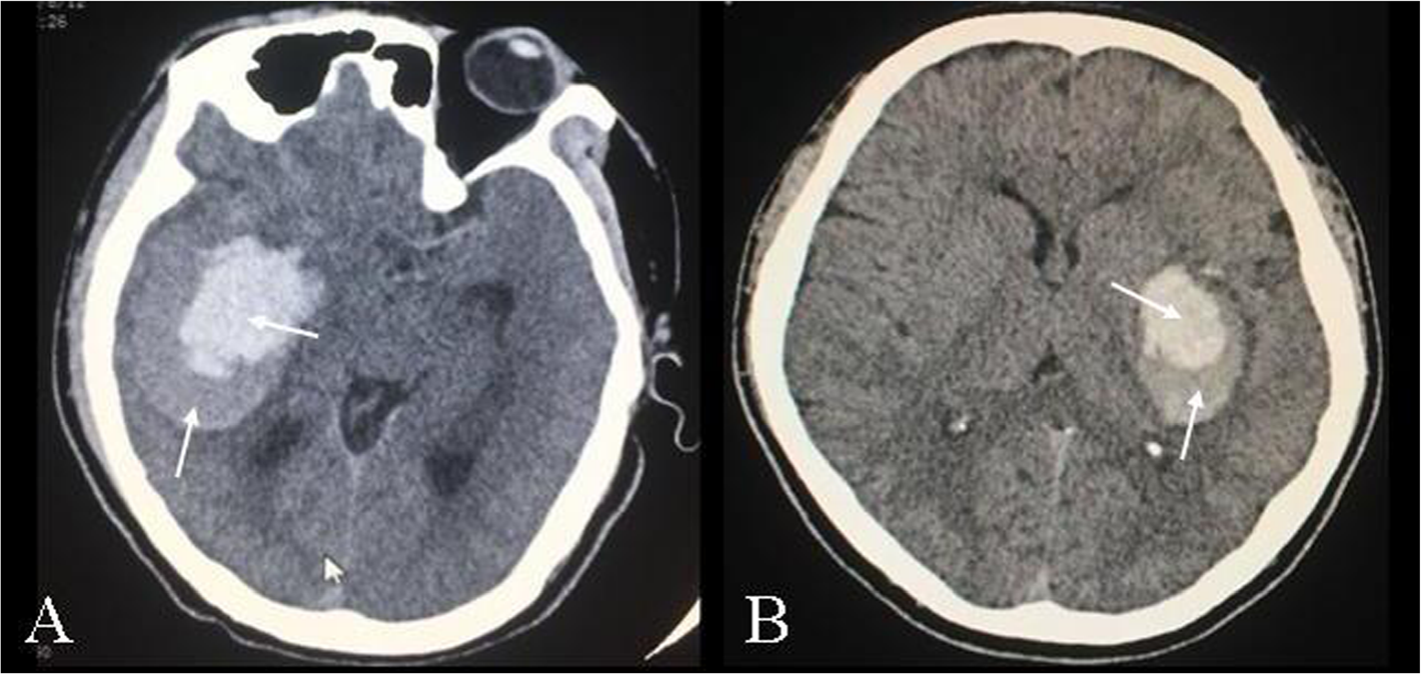
Blend signs on initial CT of patients with ICH. The ICH was located in the right (**a**) or the left (**b**) basal ganglia. The blend signs were composed of a hyperdensity and a relative hypodensity (pointed by the arrow). The boundary of the two parts was easily identified by naked eyes

CT scans (General Electric Medical Systems, Milwaukee, WI) were performed using the same parameters as in our previous studies [13]. A neurosurgical expert and a neuroimaging expert independently evaluated the ICH shape features. The haematoma shape was assessed by visual inspection [14]. The blend sign was determined by the criteria proposed in previously published studies [15]. It was composed of two parts with different densities on CT (Fig. 2).

Discrepancies about the blend sign evaluation between the two readers were resolved by discussion. The ICH volume was determined based on the method reported in a previous study [16].

### Patient treatment

#### ICH evacuation

**Fig. 3:**
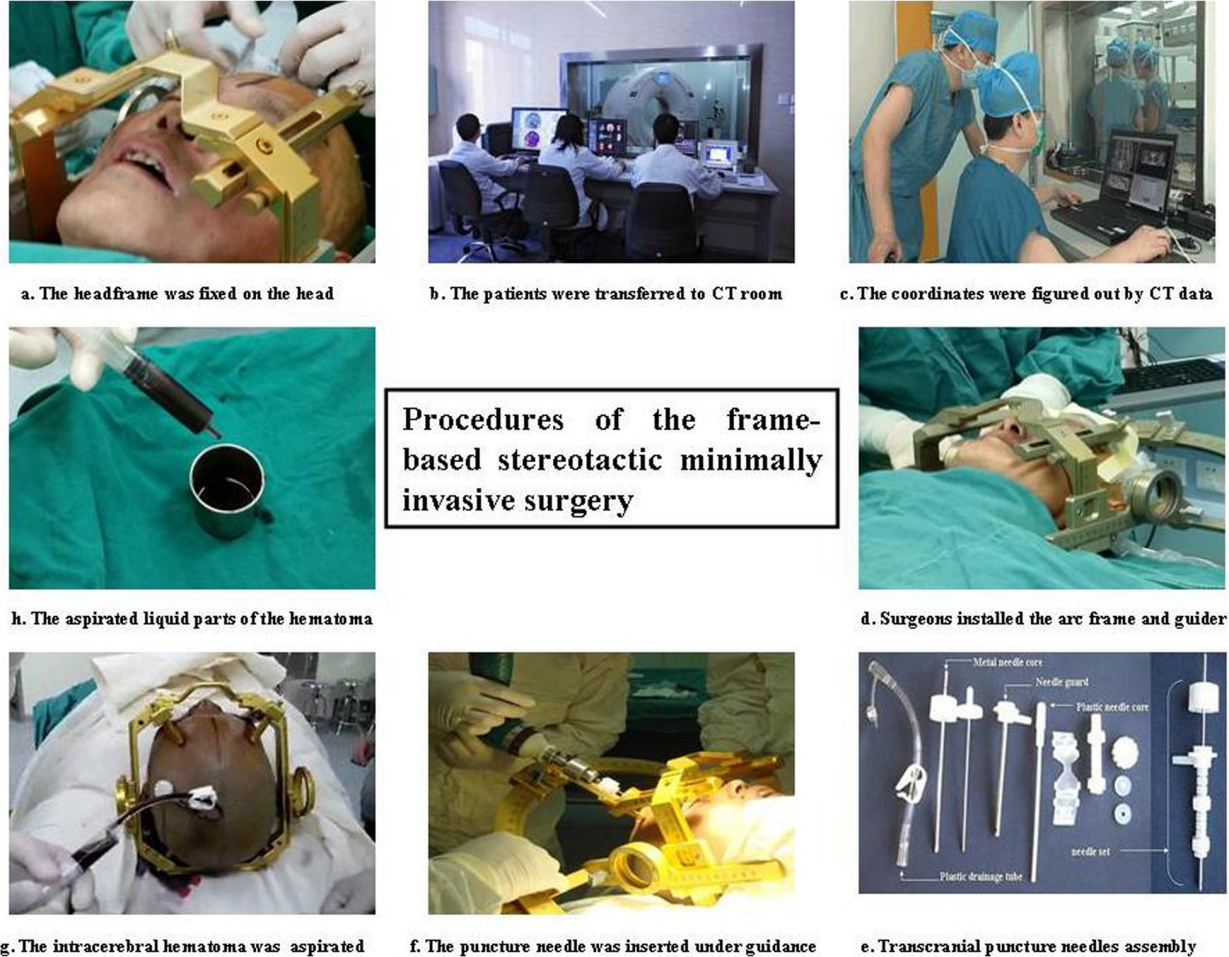
Procedures for the stereotactic minimally invasive surgery. A Positioning headframe was fixed on the head firstly and then the patient was transferred for CT scan to figure out the coordinates (**a**-**c**). Subsequently the arc frame and guider were fixed to the positioning headframe and a transcranial puncture needle was inserted (**d**-**f**). Finally, the liquid part of the ICH was aspirated out (**g**-**h**)

The surgical procedures used to remove ICH were the same as those used in our previous studies [17–19]. The skill levels of the surgeons did not affect our results, as the surgeries were performed by experienced surgeons. In brief, the patient was transferred to the operating room after a preoperative CT scan was performed. The coordinates of the ICH area were determined. Local anaesthesia at the puncture point was administered, and the skull was punctured under the guidance of a stereotactic instrument. A puncture-needle set(LY-1-type) was inserted gently into the haematoma. After removing the plastic-needle core, we used a 10-ml syringe to aspirate out the liquid part of the ICH (Fig. 3). Aspiration was stopped if 1/2 of the ICH volume was removed or resistance was encountered. We connected the needle guard to a plastic tube and retained it for drainage of the haematoma. Postoperative CT scans were performed on the first day and the third day after surgery. At any time, if a patient showed deterioration in neurological functions, another CT scan was performed.

#### Medications

All patients received standard medical management postoperatively according to the guidelines for the treatment of hypertensive ICH [14]. Measures for preventing deep venous thrombosis (DVT), controlling the body temperature and blood glucose level, supporting the patient’s nutritional state and preventing other complications were also taken [13].

### Functional outcomes

The modified Rankin Scale (mRS) score was the primary functional outcome. The mRS was administered by neurological experts blinded to the present study. The National Institutes of Health Stroke Scale (NIHSS) scores, the Glasgow Coma Scale (GCS) scores and the ICH volume changes were the secondary outcomes and obtained from medical records. The outcome was considered favourable if the patient achieved a mRS score of 0–3 points. In contrast, a poor outcome was considered if the patient achieved a mRS score of > 3 points [4].

### Complications

Severe complications, including severe pulmonary infection, respiratory failure and heart failure, were recorded and compared. Cardiopulmonary complications did not include the exacerbation of chronic heart failure, respiratory failure, or community-acquired pneumonia.

Postoperative re-haemorrhage was determined based on the criteria used in our previously published studies [12]. An increase in the haematoma volume of > 33% [20] compared to that observed in the previous follow-up CT scan was considered to indicate re-haemorrhage. Hyperdense signals that appeared again after disappearing on the most recent follow-up CT scan was also considered to indicate re-haemorrhage.

### Statistical analysis

On the basis of the assumption that 25% of patients in the blend sign group would have a mRS score of 0–3 and 45% of patients in the control group would have a mRS score of 0–3 following sMIS [4], we estimated that the inclusion of 90 patients in each group would provide 81.0% statistical power at an α level of 0.05. The permissible error *δ* was 0.1.

Commercially available software (SPSS, version 22.0) was used to analyse the differences between the blend sign group and the control group. The categorical data are expressed as proportions, and the continuous variables are expressed as means ± SDs. Student’s *t* tests (for normally distributed data) or nonparametric tests (for non-normally distributed data) were used to compare the demographic and clinical data as well as the radiological characteristics. *P* values < 0.05 indicated significant differences. The independent association between the CT blend sign and the outcomes of patients after sMIS was evaluated by binary logistic regression. κ values were calculated to assess the level of interobserver reliability. The κ values were categorized based on the criteria presented in a previous study [12].

## Results

### The baseline data

A total of 242 patients were included in our study, with ages ranging from 31 to 93 years and an average age of 57.05 ± 12.703 years. The time from onset to the baseline CT scan was 5.0 (2.0–9.7) hours. The mean GCS score at admission was 11 (8–13), and the mean NIHSS score was 16 (14–20). ICH in the basal ganglia area was detected in one hundred eighty-four patients. Thirty-four patients showed ICH in the cerebral lobes, and 24 patients showed ICH in the thalamus.

The 242 patients were assigned to the blend sign group or control group based on their haematoma features. Factors such as age, history of smoking, drinking status, preoperative ICH volume, anticoagulant use, GCS score at admission, NIHSS score at admission, time from onset to admission, time from onset to baseline CT, and time from onset to surgery did not significantly differ between the two groups. Only a higher rate of a history of hypertension was observed in the blend sign group (Table 1).

Discrepancies between the neurosurgical expert and the neuroimaging expert were noted for the scans of 3 patients. The level of interobserver agreement for identifying the CT blend sign was favourable, and the reliability was high between the 2 readers, with a κ value of 0.974 and a 95% confidence interval of 0.94–1.00.

### Changes in haematoma volume

**Fig. 4:**
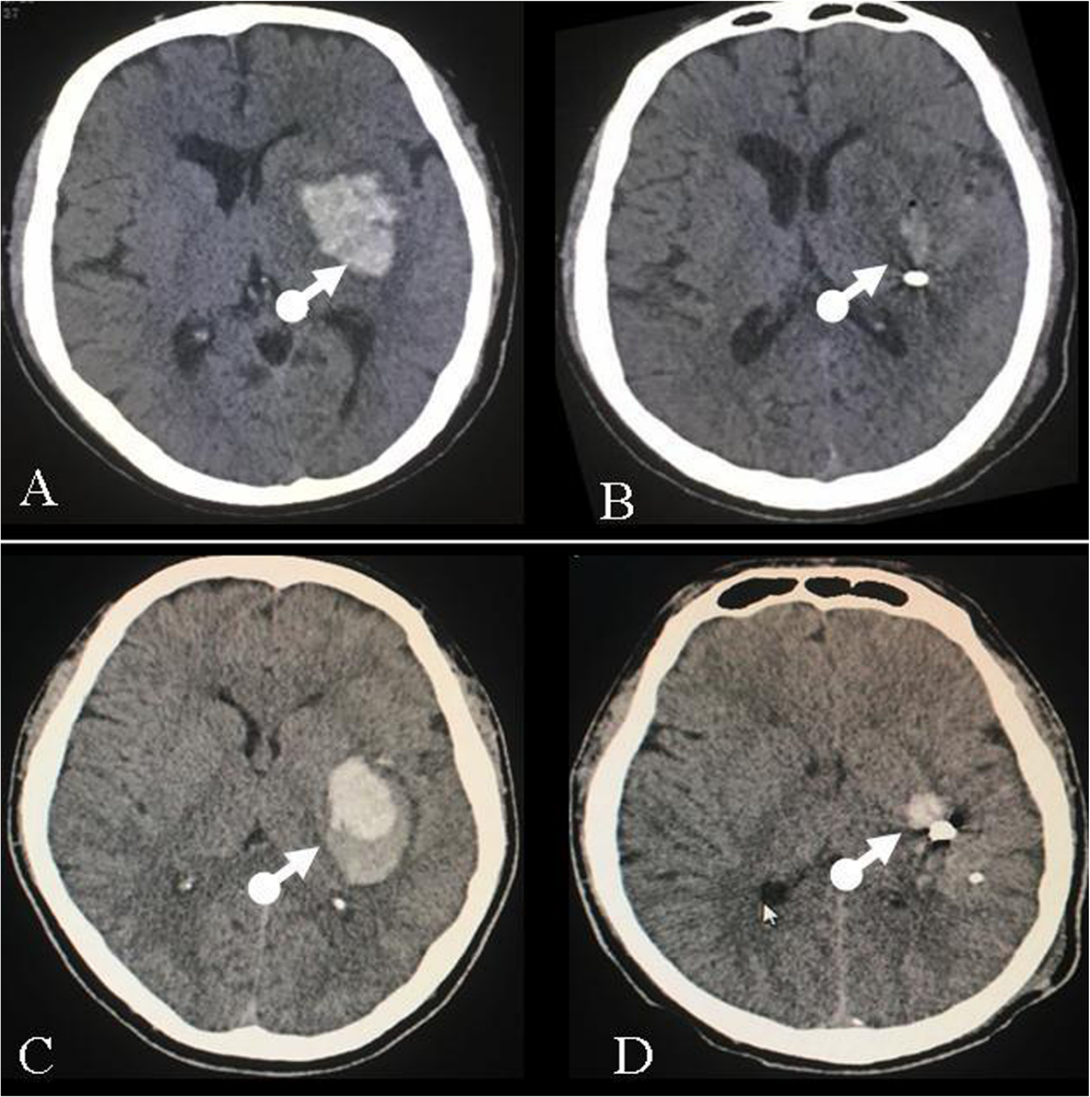
Changes in the haematoma volume after sMIS. The haematoma volume decreased significantly after the sMIS in both the patients with non-blend signs (**a**-**b**) and the patients with blend signs(**c**-**d**)

The residual ICH volume or the time to remove the drainage tube did not statistically differ between the blend sign group and the control group. No significant differences in the rates of ICH clearance were observed between the two groups (Fig. 4, Table 2). These results indicated that the blend sign had no influence on the removal of ICH by sMIS.

**Table 2:** Changes of residual haematoma volume and rate of ICH clearance during surgery

Group	Preoperative ICH volume (ml, IQR)	Postoperative Residual ICH volume (ml, IQR)	Rate of ICH clearance during surgery(%, IQR)	Time for removing the tube (days, IQR)
Blend sign group (*n* = 91)	37.8 (33–52.5)	8 (3.87–15)	30.61 (8.67–56.67)	4 (2–6)
Control group(*n* = 151)	38.0 (31–50)	8 (4.5–12)	37.27 (18.98–55.69)	4 (3–6)
*P*-value	0.379	0.648	0.809	0.904

### Changes in the GCS and the NIHSS

Both the blend sign group and the control group showed significantly increased GCS scores and decreased NIHSS scores at 1 and 2 weeks after surgery compared with those at admission (Tables 3 and Table 4). However, no significant differences were observed between the two groups at any time point. These findings suggested that the patients from the blend sign group developed the same short-term outcomes as the controls after sMIS.

**Table 3:** Changes of GCS between the blend sign group and control group (IQR)

Group	On admission (points, IQR)	One week (points, IQR)	Two weeks (points, IQR)	*P*-value
Blend sign group(n = 91)	11 (8–13)	12 (9–13)*	13 (12–15)^&^	0.013
Control group(n = 151)	11 (7–13)	12 (8–14)*	13 (9–15)^&^	< 0.001
*P*-value	0.304	0.811	0.256	

*Compared with those on admission(*P* < 0.05).^&^Compared with those on admission or with 1 week (*P* < 0.05). These results suggested that the GCS were improved 1 week after the surgery

**Table 4:** Changes of NIHSS between the blend sign group and the control group (IQR)

Group	On admission (points, IQR)	One week (points, IQR)	Two weeks (points, IQR)	*P*-value
Blend sign group(n = 91)	16 (14–20)	13 (9–17)^$^	10 (6–13)^$^	< 0.001
Control group(n = 151)	16 (13–20)	14 (10–18)^$^	12 (8–15)^$^	< 0.001
*P*-value	0.381	0.124	0.513	

^$^Compared with those on admission (*P* < 0.05). The NIHSS did not show any difference between the two groups at any time point

### Complications

Similar rates of severe complications, including pulmonary infection and heart failure, were observed between the blend sign group and the control group (*P* > 0.05, Table 5). However, a higher rate of re-haemorrhage was noted in the blend sign group (*P* = 0.049).

**Table 5:** Comparison of severe complication rate and final outcome(n,%)

Group	Pulmonary infection(n, %)	Heart failure(n, %)	Postoperative rehaemorrhage(n, %)	Good outcome(n, %)
Blend sign group(n = 91)	19 (20.9%)	2 (2.2%)	23 (25.6) &	50 (54.9%)
control group(n = 151)	30 (19.87%)	7 (4.6%)	23 (15.2)	78 (51.7%)
*P*-value	0.850	0.275	0.049	0.358

^&^Compared with the control group (*P* < 0.05). The rate of postoperative rehaemorrhage was increased compared with the control group. No significant differences were observed in the outcome between the two groups

### Influences of the CT blend sign on the outcome

Fifty patients (54.9%) from the blend sign group showed good outcomes. Among the 151 patients in the control group, 71 (51.8%) displayed good outcomes. No significant differences between the two groups were observed. Among the 128 patients with good outcomes, 50 (39.1%) showed the blend sign on the initial CT scan. The association of the CT blend sign with poor outcomes was determined by performing univariate analysis and binary logistic regression. The univariate analysis demonstrated that the CT blend sign was not statistically significantly associated with functional poor outcomes. Statistical significance (Table 6) was noted in the history of hypertension (*P* = 0.037), NIHSS score upon admission (*P* < 0.001), and GCS score upon admission (*P* < 0.001). These parameters were included in the binary logistic regression model. The final results suggested that the initial NIHSS score and the GCS score were independent predictors of poor functional outcomes in patients with ICH after sMIS (Table 7).

**Table 6:** Univariate analysis of predictors for poor outcome of patients underwent sMIS

Factors	Good outcome (128 patients)	Poor outcome(114 patients)	*P*-value
Ages(^−^x ± SD)	55.91 ± 12.55	58.43 ± 12.81	0.137
Sex (male, %)	96 (75.0%)	84 (73.7%)	0.815
History of smoking(n, %)	63 (49.2%)	57 (50.4%)	0.850
History of drinking(n, %)	51 (39.8%)	57 (50.0%)	0.113
History of hypertension(n, %)	87 (68.0%)	91 (79.8%)	0.037
Anticoagulants(n, %)	3 (2.3%)	4 (3.5%)	0.589
History of diabetes(n, %)	4 (3.1%)	9 (7.9%)	0.100
Systolic pressure (mmHg, ^−^x ± SD)	171.17 ± 26.557	176.32 ± 29.113	0.152
Diastolic pressure (mmHg, ^−^x ± SD)	101.07 ± 19.733	102.62 ± 19.676	0.541
GCS on admission (points, IQR)	12 (10–13.75)	9 (6–12))	< 0.001
NIHSS on admission (points, IQR)	16 (13–18)	17 (15–22)	< 0.001
Time from onset to baseline CT (hour, IQR)	5.0 (2.0–9.9)	4.55 (2.0–9.7)	0.764
ICH volume on admission (ml, IQR)	36 (32–50)	40 (30.75–52.39)	0.2904
Haematoma ruptured into ventricles(n, %)	43 (33.6%)	47 (41.2%)	0.220
Time from onset to surgery(h, IQR)	16 (8.13–26.75)	13.5 (10–27)	0.773
Duration of surgery (h, IQR)	1.2 (1.0–2.0)	1.5 (1.0–2.0)	0.773
Blend sign(n, %)	50 (39.1)	41 (36.0)	0.619

*GCS* Glasgow Coma Scale, *NIHSS* National Institute of Health Stroke

**Table 7:** Binary logistic regression analysis of predictors for poor outcome

Variables	B	Wals	OR	95%CI	*P*
Ages	−0.052	1.082	0.949	0.861–1.047	*0.289*
ICH volume on admission	0.033	0.510	1.033	0.945–1.130	*0.475*
History of hypertension	3.170	2.691	23.800	0.539–1.050	0.101
Anticoagulants	−0.633	0.001	0.531	0.000–106	0.085
Blend sign	0.935	0.522	212.56	0.062–7.286	0.470
GCS on admission	0.577	4.140	1.781	1.021–3.106	0.042
NIHSS on admission	0.522	4.649	1.686	1.049–2.710	0.031

Note: only the GCS and NIHSS on admission were associated with the poor outcome. The blend sign on initial CT has no effects on the outcome of patients who underwent a minimally invasive surgery

## Discussion

Spontaneous ICH accounts for 10–30% of all types of stroke worldwide. HE may predict substantially poor outcomes, and HE may be prevented if patients with high-risk factors could be distinguished in the early stage of ICH [8]. The initial CT blend sign was closely associated with poor HE outcomes in patients treated with medications [9]. The blend sign also predicted postoperative re-haemorrhage in patients with ICH after sMIS [12].

Minimally invasive procedures have been utilized in the treatment of patients with ICH in recent decades. These procedures can remove ICH with minimal iatrogenic injuries to the brain and promote the recovery of neurological functions [17, 21]. Minimally invasive procedures for ICH treatment followed by thrombolysis may be a good therapeutic strategy for ICH [22]. Minimally invasive puncture followed by medication to dissolve the clot led to the least traumatic brain injuries and the shortest operation time [4]. The initial CT blend sign predicted postoperative re-haemorrhage in sMIS-treated patients with ICH [12]. Therefore, we postulated that the blend sign could affect the outcomes of patients with ICH following sMIS. In the present study, the GCS, NIHSS, and mRS scores and postoperative complications were used as indexes to evaluate patient outcomes. However, unexpected results were observed. The GCS score increased, and the NIHSS score decreased significantly at 2 weeks after surgery compared with those at admission. However, no significant differences in the outcome were observed between the blend sign group and the control group. The proportion of patients with favourable outcomes in the blend sign group did not differ from that in the control group. In previous studies, complications after ICH were strongly associated with the outcome [18, 19]. The most common medical complication (15.1%) secondary to ICH was pneumonia [23]. Cardiac complications (5.9%) secondary to ICH also usually occur following an increase in catecholamine levels and elevated levels of brain natriuretic peptide.

In the present study, the patients in the blend sign group and non-blend sign group showed similar rates of severe pulmonary infection and heart failure following sMIS. No significant differences were observed between the two groups, suggesting that the blend sign did not influence the rate of complications following sMIS. The blend sign group showed a higher rate of postoperative re-haemorrhage than that reported in our previously published study [12]. Although the blend sign indicated poor outcomes in patients treated with medications, there was no evidence that the blend sign is associated with poor outcomes in patients after sMIS. sMIS should be performed to treat patients with the initial CT blend sign if the ICH volume is large enough and the patient is a suitable candidate for surgery.

There were some limitations of the present study. The patients were not followed up after discharge. Therefore, we were unable to determine the long-term outcomes. Some patients were discharged from the hospital without medical orders, and the death rate could not be recorded or compared, as no deaths occurred during the hospital stay. The present study was retrospective, and additional randomized prospective studies with larger sample sizes are required in the future.

## Conclusions

In conclusion, sMIS can remove intracerebral haematomas effectively. The initial CT blend sign is not associated with poor outcomes in patients with ICH following sMIS. ICH patients with the CT blend sign develop the same outcome as do patients without the CT blend sign after sMIS.

## Data Availability

The datasets analysed in the current study are available from the corresponding author upon reasonable request.

## Abbreviations

ICH: Intracerebral haemorrhage
CT: Computed tomography
sMIS: Stereotactic minimally invasive surgery
GCS: Glasgow coma scale
NIHSS: National Institute of Health Stroke Scale
